# Intensification of Polyphenol Extraction from Olive Leaves Using Ired-Irrad^®^, an Environmentally-Friendly Innovative Technology

**DOI:** 10.3390/antiox8070227

**Published:** 2019-07-18

**Authors:** Anna-Maria Abi-Khattar, Hiba N. Rajha, Roula M. Abdel-Massih, Richard G. Maroun, Nicolas Louka, Espérance Debs

**Affiliations:** 1 Centre d’Analyses et de Recherche, Unité de Recherche Technologies et Valorisation Agro-alimentaire, Faculté des Sciences, Université Saint-Joseph, B.P. 17-5208 Riad El Solh, Beirut 1104 2020, Lebanon; 2 Department of Biology, University of Balamand, Tripoli P. O. Box 100, Lebanon

**Keywords:** olive leaves, infrared-assisted extraction, response surface methodology, antioxidants, antimicrobial activity

## Abstract

Optimization of infrared-assisted extraction was conducted using Response Surface Methodology (RSM) in order to intensify polyphenol recovery from olive leaves. The extraction efficiency using Ired-Irrad^®^, a newly-patented infrared apparatus (IR), was compared to water bath (WB) conventional extraction. Under optimal conditions, as suggested by the model and confirmed experimentally, the total phenolic content yield was enhanced by more than 30% using IR as contrasted to WB, which even required 27% more ethanol consumption. High Performance Liquid Chromatography analyses quantified the two major phenolic compounds of the leaves: Oleuropein and hydroxytyrosol, which were both intensified by 18% and 21%, respectively. IR extracts increased the antiradical activity by 25% and the antioxidant capacity by 51% compared to WB extracts. On the other hand, extracts of olive leaves obtained by both techniques exhibited equal effects regarding the inhibition of 20 strains of *Staphylococcus* *aureus,* with a minimum inhibitory concentration (MIC) varying between 3.125 and 12.5 mg/mL. Similarly, both extracts inhibited Aflatoxin B1 (AFB1) secretion by *Aspergillus flavus*, with no growth inhibition of the fungus. Finally, optimization using RSM allowed us to suggest other IR operating conditions aiming at significantly reducing the consumption of energy and solvent, while maintaining similar quantity and quality of phenolic compounds as what is optimally obtained using WB.

## 1. Introduction

The cultivation of olive trees is a widespread practice in the Mediterranean region, accounting for about 98% of the world’s olive cultivation [1]. The olive tree is gradually expanding, with nearly 18 Mt of olives harvested yearly around the world. Olive tree culture and the olive processing industry produce large amounts of byproducts. It has been estimated that pruning alone produces 25 kg of byproducts (twigs and leaves) per tree, annually [2]. Olive leaves are considered to be an easily available agricultural byproduct [3,4]. They represent around 10% of the total weight of olives upon harvesting [5,6]. Olive leaves are a very rich source of bioactive compounds such as secoiridoids, flavonoids, and triterpenes [7]. They can potentially have a higher added value if their fate is reconsidered.

Valorization of the residual biomass derived from the agricultural and food sector is nowadays regarded as central to the emerging bioeconomy. This biomass is definitely underrated, despite its richness in valuable substances [8]. Olive leaves are usually disposed as waste. Otherwise, their infusion can be used in folk medicine [9]. The secoiridoid oleuropein is the main compound, along with other secoiridoids derived from tyrosol and flavonoids [5,6,9,10,11]. Other olive byproducts such as olive mill waste and mill wastewater, and wet olive pomace, have also been investigated for their polyphenols content [12].

Phenolic compounds, plant secondary metabolites, are gaining more and more interest in the agro-industrial sector. They are being extensively studied, majorly due to their biological effects. Therefore, they are the subject of numerous extraction techniques used to recover them out of their original matrices. In this regard, conventional extraction using organic solvents is the most widely used method. Nevertheless, environmental toxicity, long duration of processing, and consumption of large quantities of organic solvents are the major concerns arising from this method [13]. These major drawbacks led the researchers to seek new technologies to be applied or to be combined with pre-existing ones.

Many extraction technologies were used for the intensification of polyphenols recovery from plant materials, such as ultrasound assisted extraction [14,15], microwave assisted extraction [16,17], pressurized liquid assisted extraction [18], supercritical fluid extraction [19,20], and others. Optimization of any given extraction technique goes obviously through a maximization of polyphenols recovery while maintaining their chemical integrity and, subsequently, their functional activities.

Infrared irradiation is one of these alternatives introduced as a ‘green’ energy source [21,22] used to boost the extraction of natural products. The infrared assisted extraction apparatus Ired-Irrad^®^ is a new generation of ecofriendly machines that enhance the extraction of bioactive compounds from natural matrices using a ceramic infrared emitter [22]. Recently, this technique has been explored on pomegranate peels [14], *Prunus armeniaca* L. pomace [23], apricot pomace [24,25], and *Saussurea lappa* [26], and permitted the intensification of polyphenol recovery compared to conventional extraction methods. Infrared-assisted extraction is easy to use, economical, requires low energy consumption [23], and has a great potential to be scaled-up to an industrial level.

The innovation of this study relies on the use of a new patented technique based on infrared apparatus (IR) irradiations for the recovery of bioactive compounds, while preserving their biological properties. To our knowledge, no previous studies have investigated this effect on olive leaves. Our ultimate aim is to optimize extraction of total phenolic content from olive leaves using IR irradiation, and to compare the results with the ones obtained using water bath conventional extraction. Moreover, quality of both extracts will be inspected by testing their antioxidative and antiradical activities, their antibacterial effect against 20 strains of *Staphylococcus aureus* and 7 strains of *Escherichia coli*, and their antifungal effect not only against *Aspergillus flavus* growth, but also against its production of aflatoxin B1.

## 2. Materials and Methods

### 2.1. Plant Material

Olive leaves were provided by a local olive mill in northern Lebanon El Koura in September 2018. The leaves were washed with water to remove impurities such as dust, then dehydrated in an airflow oven at 40 °C for 48 h. Dried leaves were ground (Philips, United Arab Emirates, MEA) and then sieved using a vibrating multi sieve separator (ELE International, Loveland, CO, USA). Ground leaves, from 0.85 to 2 mm in size, were packed in plastic bags and stored at ambient temperature in the dark for further use.

#### 2.1.1. Dry Matter

Initial and final moisture contents were determined by drying the leaves for 24 h in a ventilated oven at 105 °C. The dry matter (DM) of raw material was 91 ± 0.4%.

#### 2.1.2. Chemicals

All chemicals used in the experiments were analytical grade. Folin-Ciocalteu reagent, sodium carbonate, gallic acid, 1,1-diphenyl-2-picrylhydrazyl (DPPH), 6-hydroxy-2,5,7,8-tetramethylchroman-2-carboxylic acid (Trolox), ascorbic acid, sulfuric acid, sodium phosphate, ammonium molybdate, oleuropein, and hydroxytyrosol were purchased from Sigma-Aldrich, Darmstadt, Germany.

### 2.2. Experimental Methods

#### 2.2.1. Water Bath Extraction

**Figure 1:**
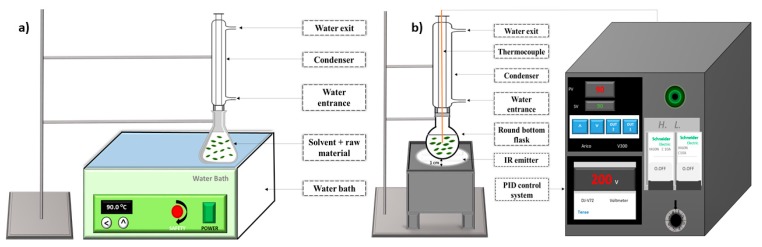
Instrumental setup for (**a**) water bath (WB) and (**b**) infrared apparatus (IR).

The conventional extraction was carried out in a digital water bath (JSR JSWB-22T, Gongju-city, Korea) (Figure 1a).

#### 2.2.2. Infrared-Assisted Extraction

The infrared-assisted extraction apparatus (Ired-Irrad^®^, Beirut, Lebanon) was designed and patented in collaboration between the faculties of Sciences of Saint-Joseph University (Beirut, city, Lebanon) and the University of Balamand (Kelhat, city Lebanon) [22]. The extraction prototype consists of a ceramic infrared emitter, linked to a proportional-integral-derivative (PID) control and temperature adjustment system. The sample consisting of olive leaves and solvent was placed in a round bottom flask connected to a condenser at a 1 cm distance from the ceramic IR emitter (Rotfil, Pianezza, Italy) (Figure 1b).

#### 2.2.3. Extraction Procedure

**Figure 2:**
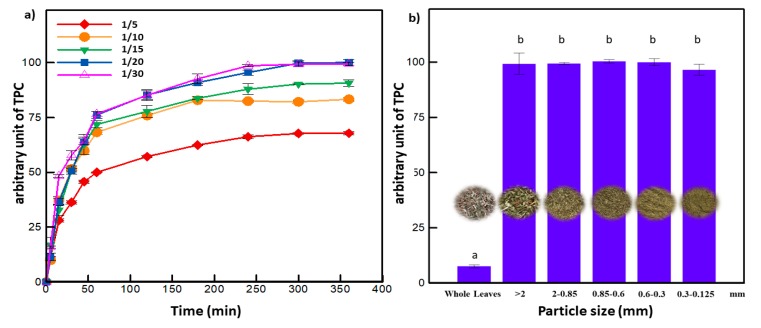
(**a**) Effect of solid to liquid ratio and (**b**) particle size on the extraction yield (letters a and b indicate significant statistical difference between means). Every arbitrary unit value is the ratio of the Total Phenolic Compounds (TPC) (at the corresponding experimental conditions) to the highest obtained TPC.

An amount of 5 g of ground leaves was added to 100 mL of solvent consisting of varying amounts of aqueous ethanol. Extractions were carried out at predetermined temperatures and time periods. The fixed particle size (0.85–2 mm) and solid to liquid ratio (1:20 *w*/*v*) were chosen based on a preliminary set of experiments (Figure 2a,b). Once the extraction was complete, the extracts were filtered through glass wool, then centrifuged for 10 min at 4500 rpm and stored at −20 °C until analyses. Prior to HPLC analyses, the supernatants were filtered using a 0.45 μm syringe after centrifugation [27].

#### 2.2.4. Total Phenolic Compounds

The total phenolic content was determined according to the Folin-Ciocalteu method [28,29]: 0.2 mL of each extract were mixed with 1 mL of ten-fold diluted Folin–Ciocalteu reagent (Sigma-Aldrich, Darmstadt, Germany), and 0.8 mL of sodium carbonate (Na_2_CO_3_) (75 g/L) (Sigma-Aldrich, Darmstadt, Germany) were added to the mixture. The absorbance was then measured by a UV-Vis spectrophotometer (Biochrom Ltd., Cambridge, England) at 750 nm. The total phenolic content was expressed as mg of Gallic Acid Equivalents per gram of dry matter mg Gallic Acid Equivalent/g DM.

### 2.3. Experimental Design

Response surface methodology (RSM) is an assemblage of statistical and mathematical methods used for products developing, improving, and optimizing processes [30]. It permits to measure the linear and quadratic effects of parameters, as well as the probable interactions between the variables.

Optimization of phenolic compounds extraction from ground olive leaves was carried out using RSM. A rotatable central composite design (2^3^ + star) (22 runs: 8 factorial design points, 6 star points and 8 center points with 12 degrees of freedom) was created to evaluate the main impact of three experimental factors: Solvent mixture, time, and temperature on the response parameter: Total Phenolic Compounds (TPC). The same design was applied twice: (1) For the extraction process using the conventional water bath (WB), and (2) for the infrared-assisted (IR) extraction apparatus. Ethanol percentage values varied between 40% and 80%, time between 60 and 180 min, and temperature between 38 °C and 77 °C (considered as −1 and +1 levels, respectively). Solvent mixture, time, and temperature are independent variables that were coded at five levels (−α, −1, 0, +1, +α). Considering three parameters and one response, experimental data were fitted to obtain a second-degree regression equation of the form:

*Y* = *β_1_* + *β_2_*E + *β_3_*t + *β_4_*T + *β_5_*E^2^ +*β_6_*t^2^ + *β_7_*T^2^ + *β_8_*Et +*β_9_*ET + *β_10_*tT (1)

where *Y* is the predicted response parameter; *β_1_* is the mean value of responses at the central point of the experiment; *β_2_*, *β_3_* and *β_4_* are the linear coefficients; *β_5_*, *β_6_* and *β_7_* are the quadratic coefficients; *β_8_*, *β_9_* and *β_10_* are the interaction coefficients; E is the solvent mixture; t is the extraction time; and T is the extraction temperature. Experimental design and statistical treatment of the results were performed using STATGRAPHICS Centurion XVII (Statgraphics 18, The Plains, Virginia).

### 2.4. High Performance Liquid Chromatography

Polyphenol (oleuropein and hydroxytyrosol) identification and quantification were conducted by HPLC, using an HPLC-DAD (diode array detection) (Waters Alliance, Milford, MA, USA), a quaternary Waters e2695 pump, an UV−vis photodiode array spectrophotometer (Waters Corporation, Milford, USA), a control system, and a data collection Empower 3 software. Analyses were carried out on a Discovery HS C18, 5 μm, 250 × 4.6 mm, column (Supelco, Bellefonte, PA, USA) with a HS C18, Supelguard Discovery, 20 × 4 mm, 5 μm, precolumn (Supelco, Bellefonte, PA, USA). The column temperature was maintained at 25 °C. Separation of 10 μL was performed at a flow rate of 0.8 mL min^−1^. Mobile phase A consisting of 0.5% (*v*/*v*) acetic acid in water and mobile phase B consisting of 100% acetonitrile were used. Solvent gradient changed according to the following conditions: From 0 to 10 min, 95% (A): 5% (B) to 70% (A): 30% (B); from 10 to 12 min, 70% (A): 30% (B) to 67% (A): 33% (B); from 12 to 17 min, 67% (A): 33% (B) to 62% (A): 38% (B); from 17 to 20 min, 62% (A): 38% (B) to 50% (A): 50% (B); from 20 to 23 min, 50% (A): 50% (B) to 5% (A): 95% (B); from 23 to 25 min, 5% (A): 95% (B) to 95% (A): 5% (B); from 25 to 35 min, 95% (A): 5% (B) to 95% (A): 5% (B). Spectrophotometric detection wavelength was carried out at 280 nm. Identification of the compounds was based on retention time of standards and comparison of spectra [31].

### 2.5. Antioxidant Activity

The total antioxidant activity of the extracts was determined using the phosphomolybdenum reduction essay [32]. The principle of this method is the formation of a green complex phosphate Mo (V). A quantity of 100 µL of each extract were mixed with 1 mL of the reagent solution (0.6 M sulfuric acid, 28 mM sodium phosphate, and 4 mM ammonium molybdate). Samples were incubated for 90 min at 95 °C. Absorbance was then measured at 695 nm. Antioxidant activity was expressed as µg of Ascorbic Acid Equivalent per milliliter (µg AAE/mL).

### 2.6. Antiradical Activity

Free radical scavenging activity was measured by the capacity of the phenolic compounds to reduce DPPH (2,2-diphenyl-picrylhydrazyl), according to [33]. 1.45 mL of DPPH (0.06 mM) (Sigma-Aldrich, St-Quentin Fallavier, France) radical was added to 50 μL of olive leaf extracts or Trolox (positive control) (Sigma-Aldrich, St-Quentin Fallavier, France). After 30 min of incubation at room temperature in the dark, the absorbance was measured at 515 nm using pure methanol as a blank. The inhibition percentage of the DPPH free radical is calculated as follows: Inhibition Percentage = [(absorbance of negative control − absorbance of sample)/absorbance of negative control] × 100. Antiradical activity was expressed as µg of Trolox Equivalent per milliliter (µg TE/mL) [34].

### 2.7. Antifungal Activity

*Aspergillus flavus* NRRL (Northern Regional Research Laboratory) 62477 isolates from spices were grown in Petri dishes containing malt extract agar (MEA) at pH 5.5 ± 0.3 for 7 days at 27 °C. A spore suspension was then prepared using Tween 80 solution. The spores were counted on a Neubauer haemocytometer (Superior, Marienfeld, Lauda-Konigshofen, Germany). The final concentration of the spore suspension was adjusted to 10^5^ spores/mL.

### 2.8. Fungal Growth Inhibition

Olive leaf extracts (125 and 250 µg) were added to the MEA medium. A final volume of 20 mL of MEA was transferred in petri dishes. For the control culture, 20 mL of MEA without polyphenols were poured in a petri dish. Afterwards, 10 µL of the previously prepared spore solution (10^5^ spores/mL) were placed in the center of each petri dish. All the dishes were left for 7 days in the incubator at 27 °C. On the seventh day, the diameters of all the cultures were measured. The *A. flavus* inhibition percentage was calculated as follows:

Inhibition % = [(Initial diameter − diameter after incubation)/initial diameter] × 100 (2)

### 2.9. Aflatoxin B1 (AFB1) Inhibition

Aflatoxin B1 (AFB1) inhibition was detected using reverse phase HPLC (diode array detection) (Waters Alliance, Milford, MA, USA) coupled with a fluorescence detector and a C18 column 5 μm, 250 × 4.6 mm, column (Supleco, Bellefonte, PA, USA) fitted with a HS C18, Supelguard Discovery, 20 × 4 mm, 5 μm, precolumn (Supelco, Bellefonte, PA, USA). The column temperature was maintained at 40 °C. The mobile phase was composed of HPLC water: Methanol: Nitric acid 4M (55:45:0.35 *v*/*v*/*v*) and 119 mg/L KBr prepared and filtered on the same day of HPLC analysis, performed with a flow rate of 0.8 mL/min. The injection volume was 100 µL, the cycle duration was 35 min, and the wavelengths for excitation and emission were 360 and 430 nm, respectively.

### 2.10. Antibacterial Activity

#### 2.10.1. Microorganisms Used

Twenty bacterial strains (American Type Culture Collections ATCC, Newman and clinical strains) of Gram-positive *S*. *aureus* and seven strains of Gram-negative *E. coli* (one *E. coli* 25921 DSM 1103 and the others are strains of different profiles of resistance) that were isolated from patients at the Centre Hospitalier Du Nord Hospital (CHN, Zghorta, Lebanon), were used in this study.

#### 2.10.2. Determination of Minimal Inhibitory Concentration for Extracts

The macro-dilution broth method was used for the determination of the minimum inhibitory concentration (MIC) of *Olea europea* extracts, as described by the Clinical and Laboratory Standards Institute [35]. A standardized bacterial inoculum was prepared and adjusted to 0.5 McFarland, then diluted to 10^6^ CFU/mL. Leaf lyophilized extracts were diluted with DMSO to produce two-fold serial dilutions ranging from 0.39 to 50 mg/mL. 1 mL of broth was added to each tube of the macro-dilution tray. 300 µL of plant extract suspension were added to the first tube in each series, after removing the same volume of broth, in order to achieve the final desired concentration. 1 mL of bacterial inoculum was added to each tube to reach 2 mL of final volume. The final extract concentration in each tube is presented in Table 1. Broth (2 mL) was used as a negative control, whereas 1 mL of Mueller-Hinton broth and 1 mL bacterial suspension were used as a positive control. The tray was then incubated for 24 h at 35 °C. Thereafter, the test tubes were checked for turbidity and MIC was determined by observing the lowest concentration of extract where there is no visible bacterial growth compared to the negative and positive control. The antibacterial analyses were repeated twice and gave the same MIC values.

### 2.11. Statistical Analysis

All experiments and measurements were done in triplicates. The mean values and the standard deviations were calculated. Error bars, in all figures, correspond to the confidence level 95%. Variance analyses (ANOVA) and Least Significant Difference (LSD) tests were done by STATGRAPHICS^®^ Centurion XV (Statgraphics 18, The Plains, Virginia).

## 3. Results and Discussion

### 3.1. Solid to Liquid Ratio and Particle Size Selection

The selection of the solid to liquid ratio and the particle size was based on primary studies (Figure 2a,b). The extraction yield increased by 50% while decreasing the solid to liquid ratio form 1:5 to 1:20 (g/mL). For a higher liquid ratio (1:30), the TPC remained constant. Hence, the solid to liquid ratio of 1:20 g/mL was adopted for all the subsequent experiments. On the other hand, lowering the particle size of olive leaves from whole leaves to 2, 0.85, 0.6, and 0.3 mm increased the contact surface area between the samples and the solvent, thus leading to a higher extraction yield. The grinding process up to 0.3 mm gave around 14 times higher recovery of polyphenols compared to whole leaves. However, for the particle size below 0.3 mm, the extraction yield decreased. Similar results were found in the literature where olive leaves of particle sizes below 0.2 mm decreased the extraction yield of polyphenols [36]. Too fine particles tend to agglomerate, limiting the accessibility of the solvent to the solid matrix and consequently altering the mass transfer phenomenon [37]. For that reason, 0.85 to 2 mm particle size was selected to be used in the following experiments.

### 3.2. Effect of Solvent Mixture, Time, and Temperature on TPC Extraction

The response surface methodology was conducted in the aim of studying and determining the optimal conditions for the highest phenolic concentration in WB and IR extracts. Based on the abovementioned results, a central composite design was conducted using RSM with the selected particle size (0.85–2 mm) and solid to liquid ratio (1:20 (g/mL)), varying solvent mixture, time, and temperature (Table 2). Values varied between 14 and 25 mg/g DM for WB and between 15 and 33 mg/g DM for IR leading to a higher TPC range with IR treatment.

**Figure 3:**
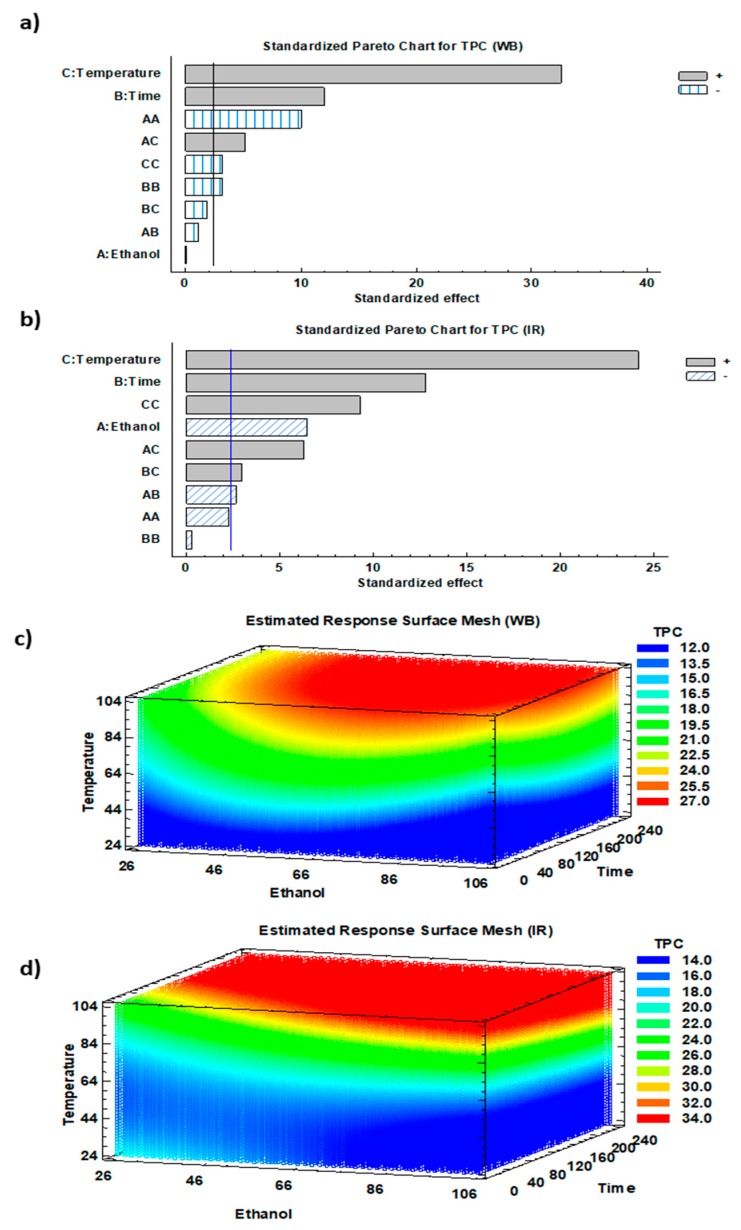
(**a**,**b**) Standardized Pareto charts and (**c**,**d**) the corresponding estimated response surface mesh of TPC for WB and IR.

The impact of the three studied parameters on the corresponding response (TPC) was analyzed according to the Pareto chart and 3D-mesh for both WB and IR (Figure 3).

Temperature and time exhibited significant linear positive effects on polyphenol extraction from olive leaves using both WB and IR (*p* < 0.01) (Figure 3a,b). Temperature rise led to a TPC increase to reach optimum at 90 °C. In addition, ethanol concentration displayed a highly significant linear negative effect in case of IR (*p* < 0.01). This might be due to the adequation existing between the used IR wavelengths and the absorption characteristics of water molecules. In this sense, the increase in ethanol percentage negatively affected the IR extraction efficiency.

Temperature had a positive quadratic effect on TPC recovery by IR. Nonetheless, a combination of linear and quadratic positive effects suggests the presence of a latency phase followed by a fast increase in the variation of TPC as a function of temperature. For WB extraction, and despite that ethanol displayed a non-significant effect, it exhibited a quadratic significant negative effect on TPC (Figure 3a,b). This observation implies that the majority of the extracted phenolic compounds of olive leaves were recovered with an intermediate solvent polarity varying between that of pure water and pure ethanol. Previous studies discussed the interaction between the IR wavelength emitted and its capacity of being absorbed by the solvent, which is in relation with the solvent polarity. The elemental and most important features of infrared radiation are the high heat transfer capacity, heat penetration straight into the product, fast management response, and good chances for process control [38].

Ethanol time interaction had a positive effect (*p* < 0.05) on TPC, with a higher impact observed using IR (Figure 3b). Although ethanol played a negative linear effect on TPC, it positively amplified the positive linear effect of temperature. Other mildly significant interactions exist in the case of IR.

Finally, 3D mesh diagrams showed the spectrum of phenolic compounds’ yields simultaneously as a function of solvent mixture, time, and temperature (Figure 3c,d). The red areas highlight the ranges of the highest TPC reached with different combinations of the three operating conditions.

Increasing both time and temperature led to an increase in the TPC in both WB and IR extracts, giving a maximum recovery at 90 °C. Diffusion coefficient is well known to be directly proportional to temperature elevation [39]. Furthermore, increasing the extraction time at a constant temperature also appeared to heighten TPC. This phenomenon is explained by Fick’s second law of diffusion that predicts a final equilibrium between the solute concentrations in the solid matrix and in the extraction solution after a certain time [40].

### 3.3. Optimization of TPC Extraction

WB optimum TPC yield was reached with 70% ethanol/water, 193 min, and 90 °C, while IR optimum was reached with 55% ethanol/water, 220 min, and 90 °C (Table 3). Maximum extraction yields for the optimum conditions were 27.12 and 36.23 mg GAE/g DM for WB and IR, respectively. These suggested optimums reflect the operating conditions required for the recovery of the highest polyphenol content in both WB and IR.

Response values were given by statistical analysis to fit the model equation (Table 4). The regression equation allowed for calculating the TPC predicted values that will be lately compared to the experimental ones.

In order to verify the predictive capacity of the model, optimum conditions were revalidated and obtained values (26.31^a^ ± 0.8 and 34.28^b^ ± 1.9 mg/g DM) were compared to the values predicted by the model (Table 5). Predicted values displayed a great correlation with experimental values and resulted in the calculation of the coefficients of determination (*R^2^*) which are 95% and 90% for WB and IR respectively.

A comparison between both optima was the main objective of this response study. The maximum amounts of TPC were obtained at an ethanol concentration of 70% and 55% for WB and IR, respectively. This is in accordance with previous investigations, which indicated that the conventional extraction of biophenols from olive leaves with water/ethanol solutions peaked at 70% ethanol [27]. The recovery of polyphenols from olive leaves was also optimized by pressurized liquid extraction. The optimal conditions were found to be: 50% ethanol/water mixture at 80 °C, with 2 cycles of 5 min giving a TPC of 53.15 mg GAE/g [41]. Furthermore, 80% ethanol [42] and 60% ethanol [43] gave the highest yield using ultrasound-assisted extraction in precedent studies. Compared to these ultrasound extractions, IR permitted the reduction of ethanol consumption, which is of great economic interest when extended to an industrial level. In addition, reducing the use of organic solvent consumption limits its related environmental damage and is considered to be one of the most important principles of green chemistry. Organic solvents react in the atmosphere under sunlight, producing air pollutants that seriously affect human, animal, and plant health. To this end, a very important future challenge resides in the development of new environmentally-friendly analytical methods capable of giving good quality results [44].

Many studies evaluated the phenolic composition of the enhanced recovery of olive leaf extracts by different technologies. Analogous amounts (24.36 ± 0.85 mg GAE/g DM) were reached with conventional extraction in a previous study using the same ethanol/water concentration [45]. Obtained results were also comparable to those found earlier for similar extracts, recovered with ultrasound-assisted extraction (25.06 mg GAE/g DM [46], 38.66 mg GAE/g DM [47]). In another study, ultrasound showed an improvement in the extraction yield compared to conventional extraction [48]. Also, resembling yield (37.52 ± 0.87 mg GAE/g DM) was attained using subcritical water extraction but while using 56% higher temperature and 60% more solvent usage. Likewise, microwave-assisted extraction was studied and compared to conventional method: 55% extraction yield amelioration compared to WB using 80% methanol/water [31].

The suggested IR optimum of TPC obtained by the RSM study can be further reconsidered in order to fit different industrial or environmental demands. For example, in order to reach with IR technology, the same optimal TPC (27.12 mg GAE/g DM) acquired by WB, ethanol could be reduced to 12%, temperature to 40 °C, and time raised to 240 min.

In the subsequent sections, extractions were carried out in the optimum conditions using WB and IR and used in the upcoming analyses.

### 3.4. High Performance Liquid Chromatography

It has been frequently reported that oleuropein and hydroxytyrosol are considered as major phenolic compounds of olive leaves [49,50]. HPLC analyses were done for both WB and IR optimum extracts in order to compare oleuropein and hydroxytyrosol contents, and then to correlate these results with the TPC values.

As shown in Table 6, optimum conditions led to highest values of oleuropein and hydroxytyrosol contents in IR extract of 14.01^b^ ± 0.9 and 0.40^d^ ± 0.008 mg/g DM, while 11.84^a^ ± 1.2 and 0.33^c^ ± 0.02 mg/g DM in WB extract, respectively. In a previous investigation, it was reported that the oleuropein yield was 6.53 ± 0.01 mg GAE/g DM (2.15 times lower than our results), and hydroxytyrosol 0.54 ± 0.02 mg GAE/g DM (almost the same amount as our findings), following an ultrasonic extraction for 3 min at 50 °C using 75% ethanol/water [51]. These valuable compounds in olive leaf extracts are responsible for many health benefits. Oleuropein is reported to have antimicrobial activities against viruses, retroviruses, bacteria, yeasts, fungi, and other parasites [52]. On the other hand, hydroxytyrosol is known to be beneficial for treating atherosclerosis and prevents diabetic neuropathy [53]. In particular, both molecules have demonstrated high antioxidant and antimicrobial activities [54]. Therefore, there is a growing interest to use these bioactive molecules in various industrial applications in food supplements in the pharmaceutical and cosmetic industries [55]. Antioxidant, antiradical, antibacterial, and antifungal activities of the two optimum extracts were thus compared.

### 3.5. Antioxidant and Antiradical Activity

**Figure 4:**
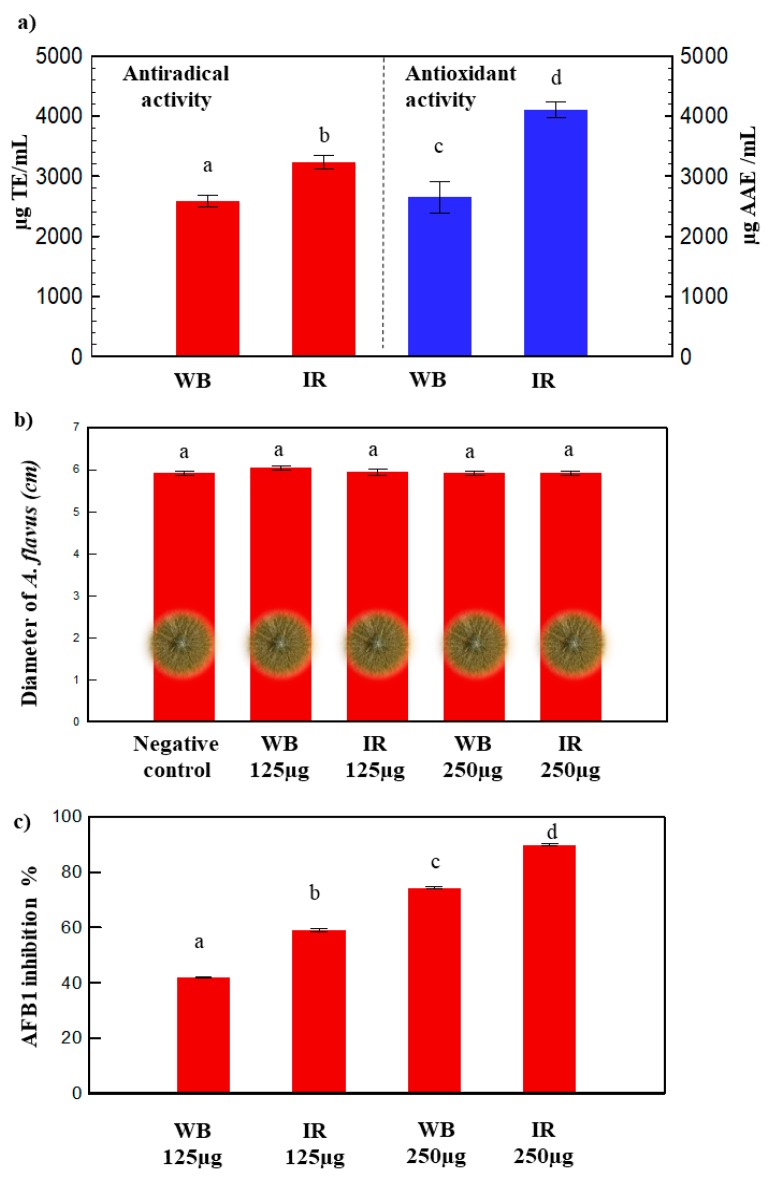
(**a**) Antiradical and antioxidant activities, (**b**) fungal growth of *A. flavus* and (**c**) AFB1 inhibition percentage for WB and IR extracts (a, b, c, and d indicate significant statistical difference between means).

Two methods were used to assess the antioxidant and antiradical capacities of olive leaf extracts: The phosphomolybdenum and the DPPH assays (Figure 4a).

IR extract gave the greatest antioxidant activity 4002.94^a^ ± 24 µg AAE/mL compared to WB 2653.23^b^ ± 263 µg AAE/mL (Figure 4a). The IR extract also exhibited the highest antiradical activity 3237^a^ ± 115 µg TE/mL compared to that of WB: 2589^b^ ± 93 µg TE/mL. A previous study displayed a lower antiradical activity of about 1930 ± 0.09 µg TE/mL, using ultrasound-assisted extraction of polyphenols from olive leaves at 50 °C with 75% ethanol/water [51].

### 3.6. Antifungal Activity

The phenolics obtained by the optimal extraction conditions of WB and IR did not affect the fungal growth, but significantly inhibited the AFB1 production by *A. flavus* (Figure 4b,c). The observed inhibition percentages of the toxin varied between 40% and 90%, with the increase of the polyphenol concentration of the extract from 125 µg to 250 µg (Figure 4c). Both WB and IR extracts were able to disrupt the AFB1 production by *A. flavus*, without having any significant effect on the fungal growth (Figure 4b,c). However, the impact of IR on the inhibition of aflatoxin B1 biosynthesis was ≈1.3 times higher compared to WB. This reflects the synergetic effect of phenolic compounds present in each extract that led to a modified biological efficiency for WB and IR, even at the same polyphenol concentration. The synergistic effects of phenolics against the growth of *A. flavus* using IR was also observed for pomegranate peel extracts [14]. AFB1 is known to be the most predominant and toxic aflatoxin. It is also known as being one of the mainly genotoxic agents and hepatocarcinogens identified [56]. It is very interesting to be able to inhibit the mycotoxin secretion without affecting the fungal growth and modifying the microbial ecology of the crop.

### 3.7. Antibacterial Activity

Different studies claim strong antimicrobial activity of oleuropein and hydroxytyrosol against many bacteria (e.g. *Bacillus cereus, Listeria monocytogenes,* etc.) [17,57,58]. In the present study, WB and IR olive leaf extracts showed antibacterial activity against all the tested *S. aureus* strains, with mean MIC values that range between 3.125 and 12.5 mg/mL (Table 1). The lowest MIC was recorded for *S. aureus 004* and *S. aureus 005* as 3.125 mg/mL. Both WB and IR extracts exhibited similar antibacterial activity suggesting that active compound(s) were not affected by the IR extraction method. Moreover, the IR technology ameliorated the antioxidant and antiradical activities, as well as it inhibited the AFB1 production (Figure 4).

For all the tested concentrations of polyphenols (0.39; 0.78; 1.56; 3.12; 6.25; 12.5; 25; and 50 mg/mL), no antibacterial activity against *E. coli* strains was detected.

Similarly, olive leaf extracts obtained with mechanical stirring for 12 hours, using acetone, did not show any antibacterial activity against *E. coli* (ATCC 25922); however, an MIC of 2.5 mg/mL against *S. aureus* (ATCC 25923) was observed [59]. This might be due to the fact that Gram-negative bacteria possess an outer membrane that acts as a barrier to many environmental substances [60].

## 4. Conclusions

This study revealed the efficiency of Ired-Irrad^®^ technology for the intensification of polyphenol recovery from olive leaves. Time and temperature were shown, by response surface methodology, to be the most significantly-affecting infrared-assisted extraction parameters. Compared to the conventional methods, IR allowed the extraction of a higher yield of polyphenols and improved many of their biological activities—i.e., antioxidant, antiradical, and anti-AFB1 secretion. Compared to WB, IR technology enhanced the recovery of both oleuropein and hydroxytyrosol, the two main polyphenols present in olives leaves. IR seems to be a very promising new generation of ecofriendly machines that can enhance the extraction of polyphenols with less energetic and solvent consumptions.

## 5. Patents

Rajha, H.N., Debs, E., Maroun, R.G., Louka, N. (2017). System for extracting, separating or treating products through infrared radiation. Adequacy between the properties of infrared radiation and those of the processed products. Invention patent number 2017 / 11-11296L granted on 29/11/2017.

## Figures and Tables

**Table 1:** Minimum Inhibitory Concentrations (MICs) of WB and IR olive leaves extract against twenty *Staphylococcus aureus* strains.

Bacterial Strain	Olive Leaves Extract Minimum Inhibitory Concentrations (mg/mL)	
	WB	IR
*S*. *aureus 001*	12.5	12.5
*S. aureus 002*	6.25	6.25
*S. aureus 003*	12.5	12.5
*S. aureus 004*	3.125	3.125
*S. aureus 005*	3.125	3.125
*S. aureus 006*	12.5	12.5
*S. aureus 007*	12.5	12.5
*S. aureus 008*	12.5	12.5
*S. aureus 009*	12.5	12.5
*S. aureus 010*	12.5	12.5
*S. aureus 011*	12.5	12.5
*S. aureus 012*	12.5	12.5
*S. aureus 013*	12.5	12.5
*S. aureus 014*	12.5	12.5
*S. aureus 015*	12.5	12.5
*S. aureus 016*	12.5	12.5
*S. aureus 017*	12.5	12.5
*S. aureus Newman*	6.25	6.25
*S. aureus ATCC 29213*	6.25	6.25
*S. aureus N315 MRSA*	12.5	12.5

**Table 2:** Arrangement for independent variables and their responses for TPC (mg GAE/g dry matter (DM)).

Run	Central Composite Design	Variable Levels Uncoded			Phenolic Compounds Yield (mg GAE/g DM)			
		Solvent (% Ethanol)	Time (min)	Temperature ( °C)	WB		IR	
					Experimental	Predicted	Experimental	Predicted
1	Factorial design points	40	60	38	15.74	15.00	19.71	18.27
2		80	60	38	14.41	13.81	16.61	14.73
3		40	180	38	18.35	18.47	23.20	22.40
4		80	180	38	16.12	16.65	18.39	16.63
5		40	60	77	22.52	21.16	21.02	22.15
6		80	60	77	23.90	22.94	23.66	23.81
7		40	180	77	23.81	23.58	27.51	28.75
8		80	180	77	24.81	24.72	27.38	28.18
9	Star points	26.36	120	57.5	17.41	18.33	21.90	21.51
10		93.63	120	57.5	18.04	18.30	16.76	18.06
11		60	19.09	57.5	16.31	18.09	16.46	17.37
12		60	220.9	57.5	23.11	22.51	23.86	23.86
13		60	120	24.7	14.31	14.31	15.01	18.20
14		60	120	90.3	25.11	26.28	33.47	31.18
15		60	120	57.5	21.20	21.22	21.77	20.81
16	Center points	60	120	57.5	21.15	21.22	20.17	20.81
17		60	120	57.5	21.43	21.22	20.32	20.81
18		60	120	57.5	21.09	21.22	20.70	20.81
19		60	120	57.5	21.20	21.22	20.45	20.81
20		60	120	57.5	21.49	21.22	20.51	20.81
21		60	120	57.5	20.47	21.22	21.29	20.81
22		60	120	57.5	21.89	21.22	21.45	20.81

**Table 3:** Optimum extraction conditions for WB and IR.

Factor	Optimum Conditions	
	WB	IR
Ethanol/Water (%)	70.16	55.35
Time (min)	193.28	220.91
Temperature ( °C)	90.29	90.29

**Table 4:** Second order regression equation for TPC of each extraction technique and the R-squared of each equation.

Extraction Technique	*R^2^* (Percent)	Equation
WB	90.69	TPC = 29.1281 − 0.0854381E + 0.0347351t − 0.487806T − 0.000850084E^2^ − 0.0000128046t^2^ + 0.00366906T^2^ − 0.00046598Et + 0.00333862ET + 0.000528357tT
IR	95.23	TPC = −1.18369 + 0.214169E + 0.0644087t + 0.193729T − 0.0025667E^2^ − 0.0000897562t^2^ − 0.000854751T^2^ − 0.000132978Et + 0.0019014ET − 0.000225784tT

**Table 5:** Predicted and experimental results of TPC (mg GAE/g DM) for WB and IR.

Optimum TPC Value (mg GAE/g DM)	Predicted	Experimental
WB	27.12	26.31^a^ ± 0.3
IR	36.23	34.28^b^ ± 1

a and b indicate significant statistical difference between means.

**Table 6:** Oleuropein and hydroxytyrosol concentrations (mg/g DM) in WB and IR olive leaf extracts.

Concentration (mg/g DM)	Extraction Technique	
	WB	IR
Oleuropein	11.84^a^ ± 1.2	14.01^b^ ± 0.9
Hydroxytyrosol	0.33^c^ ± 0.02	0.40^d^ ± 0.008

Different letters (a, b, c, and d) indicate significant statistical difference between means.
